# The moderating role of childhood adversity subtypes on the relationship between autistic traits and psychotic experiences in the general population

**DOI:** 10.1192/j.eurpsy.2025.328

**Published:** 2025-08-26

**Authors:** M. Karaçam Doğan, T. Prachason, B. Lin, L.-K. Pries, A. Arias-Magnasco, R. Bortoletto, C. Menne-Lothmann, J. Decoster, R. van Winkel, D. Collip, P. Delespaul, M. De Hert, C. Derom, E. Thiery, N. Jacobs, J. van Os, B. Rutten, N. Brondino, M. Colizzi, L. Fusar-Poli, S. Guloksuz

**Affiliations:** 1 Department of Psychiatry, Kdz Eregli State Hospital, Zonguldak, Türkiye; 2 Department of Psychiatry, Mahidol University, Bangkok, Thailand; 3 Department of Psychiatry and Neuropsychology, School for Mental Health and Neuroscience, Maastricht University, Maastricht, Netherlands; 4 Unit of Psychiatry, University of Udine, Udine, Italy; 5 University Psychiatric Centre, KU Leuven; 6 Centre of Human Genetics, University Hospital Leuven, Leuven; 7 Department of Neurology, University of Gent, Gent, Belgium; 8 Faculty of Psychology and Educational Sciences, Open University of the Netherlands, Heerlen; 9 University Medical Centre Utrecht, Brain Centre Rudolf Magnus, Utrecht, Netherlands; 10 Department of Psychosis Studies, Institute of Psychiatry, King’s College London, London, United Kingdom; 11 Department of Brain and Behavioral Sciences, University of Pavia, Pavia, Italy; 12 Department of Psychiatry, Yale School of Medicine, New Haven, United States

## Abstract

**Introduction:**

Childhood trauma (CT) and bullying have been shown to be associated with autistic traits (AT) and to mediate the relationship between AT and psychotic experiences (PEs). Understanding how different childhood adversity subtypes influence distinct domains of PEs can provide important insights for preventing PEs in individuals with high AT.

**Objectives:**

This study aimed to investigate the impacts of childhood adversity subtypes on the relationship between AT and PEs in a general population twin sample.

**Methods:**

We analyzed data on 792 twins and siblings from the first wave of the TwinssCan Project, a longitudinal general population twin cohort. AT were measured using the Autism-Spectrum Quotient (AQ), and PEs using the Community Assessment of Psychic Experiences (CAPE) which consists of three subscales (positive, negative and depressive). CT subtypes were assessed using the Childhood Trauma Questionnaire (CTQ), evaluating emotional, physical, and sexual abuse, as well as emotional and physical neglect. Bullying was assessed using the Retrospective Bullying Questionnaire (RBQ). Multilevel linear regression analyses were conducted. Positive, negative, and depressive PEs were regarded as the dependent variables, interactions of CT subtypes and bullying with total AQ were regarded as independent variables in separate models. All models were adjusted for age and sex. Bonferroni correction (p<0.008) was applied for multiple comparisons.

**Results:**

The mean of the age of the participants was 17.5 ± 3.6 years and 60.2% of the sample was female. Emotional abuse (B = 0.12, 95% CI: 0.08 to 0.15), physical abuse (B = 0.15, 95% CI: 0.07 to 0.22), sexual abuse (B = 0.10, 95% CI: 0.03 to 0.16), emotional neglect (B = 0.05, 95% CI: 0.02 to 0.09) and physical neglect (B = 0.07, 95% CI: 0.03 to 0.11) significantly moderated the positive relationship between AT and positive PEs. However, only emotional abuse uniquely moderated the relationship between AT and both negative and depressive PEs (B = 0.09, 95% CI: 0.04 to 0.13; B = 0.09, 95% CI: 0.04 to 0.14) with physical neglect showing a trend toward significance for depressive PEs (B = 0.08, 95% CI: 0.02 to 0.13). Bullying did not moderate any domain of PEs.

**Conclusions:**

Emotional abuse may play a unique role in moderating the relationship between AT and PEs, affecting all domains of psychosis expression. Conversely, other forms of CT predominantly influence positive PEs. Further research is needed to clarify the distinct pathways through which distinct types of childhood adversity impact the association between AT and PEs.

**Disclosure of Interest:**

M. Karaçam Doğan: None Declared, T. Prachason: None Declared, B. Lin: None Declared, L.-K. Pries: None Declared, A. Arias-Magnasco: None Declared, R. Bortoletto: None Declared, C. Menne-Lothmann: None Declared, J. Decoster: None Declared, R. van Winkel: None Declared, D. Collip: None Declared, P. Delespaul: None Declared, M. De Hert: None Declared, C. Derom: None Declared, E. Thiery: None Declared, N. Jacobs: None Declared, J. van Os: None Declared, B. Rutten: None Declared, N. Brondino : None Declared, M. Colizzi Consultant of: GW Pharma Limited, GW Pharma Italy SRL and F. Hoffmann-La Roche Limited, outside of this work. , L. Fusar-Poli: None Declared, S. Guloksuz: None Declared

